# Atomic structures, conformers and thermodynamic properties of 32k atmospheric molecules

**DOI:** 10.1038/s41597-023-02366-x

**Published:** 2023-07-12

**Authors:** Vitus Besel, Milica Todorović, Theo Kurtén, Patrick Rinke, Hanna Vehkamäki

**Affiliations:** 1 University of Helsinki, Institute for Atmospheric and Earth System Research, Helsinki, 00014 Finland; 2 University of Turku, Dept. Mechanical and Materials Engineering, Turku, FI-20014 Finland; 3 Aalto University, Dept. of Applied Physics, P.O. Box 11100, FI-00076 Aalto Espoo, Finland

**Keywords:** Atmospheric science, Environmental sciences, Theoretical chemistry

## Abstract

Low-volatile organic compounds (LVOCs) drive key atmospheric processes, such as new particle formation (NPF) and growth. Machine learning tools can accelerate studies of these phenomena, but extensive and versatile LVOC datasets relevant for the atmospheric research community are lacking. We present the GeckoQ dataset with atomic structures of 31,637 atmospherically relevant molecules resulting from the oxidation of *α*-pinene, toluene and decane. For each molecule, we performed comprehensive conformer sampling with the COSMO*conf* program and calculated thermodynamic properties with density functional theory (DFT) using the Conductor-like Screening Model (COSMO). Our dataset contains the geometries of the 7 Mio. conformers we found and their corresponding structural and thermodynamic properties, including saturation vapor pressures (*p*_*Sat*_), chemical potentials and free energies. The *p*_*Sat*_ were compared to values calculated with the group contribution method SIMPOL. To validate the dataset, we explored the relationship between structural and thermodynamic properties, and then demonstrated a first machine-learning application with Gaussian process regression.

## Background & Summary

With climate change accelerating, humanity faces unprecedented social, ecological and economic changes^1^. While data-driven research is emerging in atmospheric science^2–5^, open research data is not yet as readily available as in many other fields^6–10^. We present our contribution to data-driven atmospheric science in form of the GeckoQ dataset that provides molecular data relevant for aerosol particle growth and formation.

Aerosol particles and clouds affect the climate by absorbing and reflecting sunlight in the atmosphere, but their impact on global warming is still poorly understood^11^. Aerosol particles can also act as cloud condensation nuclei. They are either emitted or grow from gaseous molecules in the atmosphere, a process known as *new particle formation* (NPF). Estimates make NPF responsible for 40–70% of all cloud condensation nuclei^12^. Recently, organic molecules have been identified as major contributors to initial aerosol particle growth and formation up to sizes where the particles can act as condensation nuclei^13–16^. A key molecular property related to aerosol particle growth is the saturation vapor pressure (*p*_*Sat*_), a measure for a molecule’s ability to condense to the liquid phase. Thus, molecules with a low *p*_*Sat*_, *low-volatile organic compounds* (LVOC), are of special interest for NPF research. However, LVOC are difficult to study experimentally as there are millions of potential LVOC structures in the atmosphere. Due to the large number of LVOC species and their low volatilities, the gas phase concentration of any single compound is often far below the instrumental detection limit^17^.

Computational tools, such as density-functional theory (DFT) offer a complementary approach to study LVOCs^18–20^. However, due to its computational expense, DFT has not yet been widely used to generate datasets in atmospheric science. Wang *et al*.^21^ compiled a dataset of 3414 molecules extracted from the the Master Chemical Mechanism^22–24^. They computed the saturation vapour pressure (*p*_*Sat*_) on the same level of theory used in this work, but the dataset size is relatively small for meaningful machine learning^25,26^. Krüger *et al*.^5^ trained deep learning models on 103,040 quinones, but did not extend their study beyond this single molecular class. Finally, Isaacman-VanWertz and Aumont^27^ studied the *p*_*Sat*_ of 182,000 atmospheric species with computationally-efficient group contribution methods, but did not apply more accurate DFT methods. The dataset presented in this article is derived from the latter study: we extend it with rigorous conformer search and thermodynamic calculations.

In this article, we introduce the **GeckoQ** dataset encompassing carefully-curated 31,637 LVOCs. To ensure atmospheric relevance, we employed the chemical mechanism GECKO-A^28^, that simulates the oxidation of hydrocarbon emissions (in the following referred to as *parent species*) to generate molecules following previous work^27^. To provide an accurate *p*_*Sat*_, we used a well established approach of conducting a conformer search with the COSMO*conf* program, and then we calculated *p*_*Sat*_ with the COSMO*therm* program^21,29,30^. For each molecule, GeckoQ features important thermodynamic properties: **saturation vapor pressures** [Pa] (*p*_*Sat*_), the **chemical potential** [kJ/mol], the **free energy of molecule in mixture** [kJ/mol], and the **heat of vaporisation** [kJ/mol] calculated with DFT. GeckoQ also contains the optimized geometries of all conformers that were included in the calculations, summing up to 7,259,598 structures and associated total energies for the whole dataset, thereby exceeding even the NablaDFT dataset^31^ in size.

**Fig. 1:**
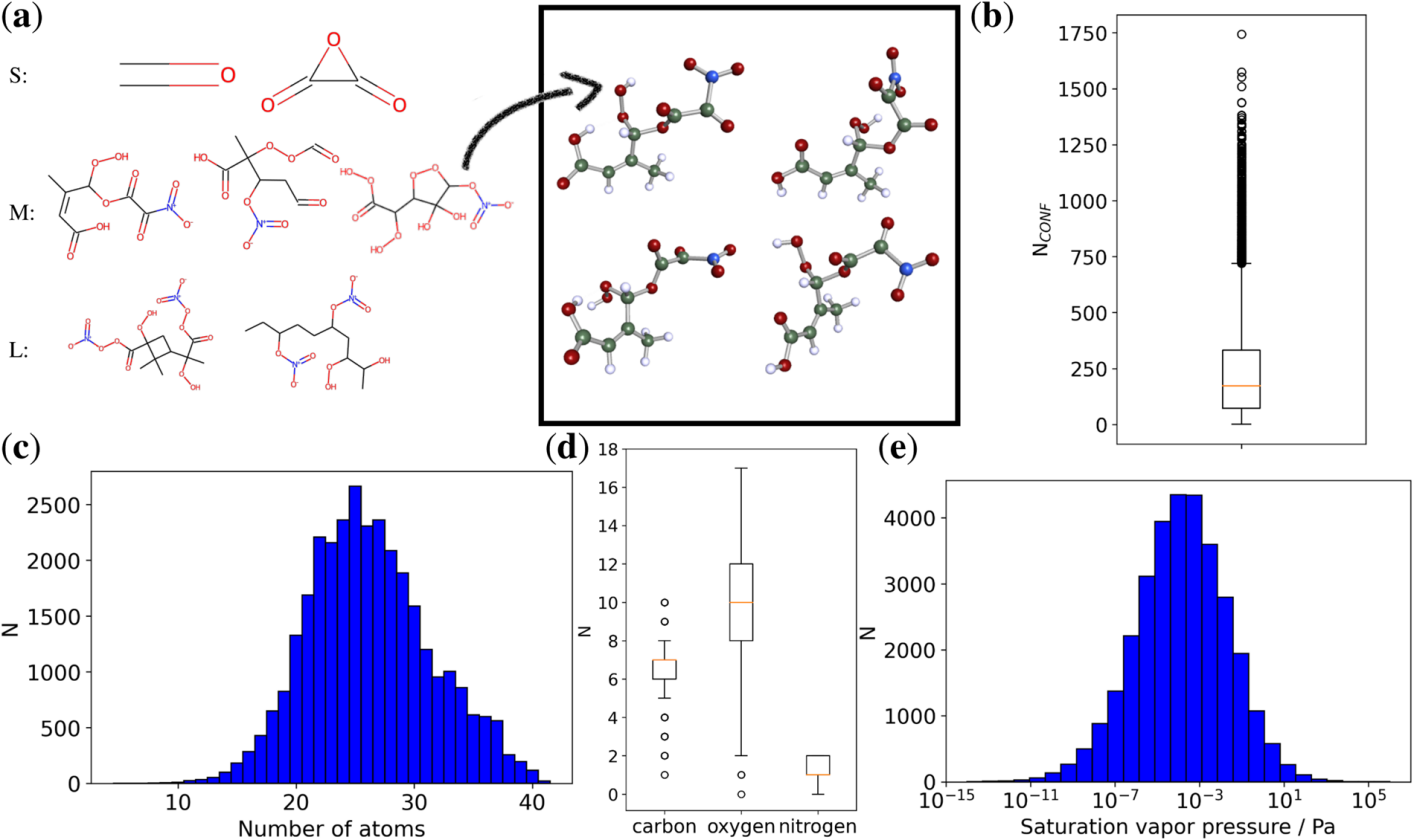
A general overview of the data: (**a**) Sample molecules for small (S), medium (M) and large (L) sizes in terms of number of atoms. For one M sized molecule four conformers of overall 140 conformers are depicted, with carbon in green, nitrogen in blue, oxygen in red and hydrogen in blue. (**b**) A boxplot of the number of conformers found per molecule (median 173). (**c**) The distribution of the molecule size in terms of the number atoms. (**d**) Boxplots for the different atomic species present in the data, excluding hydrogen. (**e**) The histogram of the *p*_*Sat*_ values in the data.

Figure 1 presents a general overview of the GeckoQ dataset. Panel (a) depicts typical GeckoQ molecules. They consist of carbon backbones derived from the parent species decane, toluene and *α*-pinene to which various functional groups are attached. In some cases ring structures persist from the original *α*-pinene and toluene, but also new rings involving oxygen have formed. The number of conformers depends on the number of functional groups and the length of the carbon backbone, and thus on the size of the molecule. The median number of conformers per molecule is 173, but we found up to 1750 conformers for a single molecule (see Fig. 1b). The molecular size distribution in GeckoQ (cf. Figure 1c) peaks around 25 atoms and is slightly skewed towards larger molecules. The smallest molecule is formaldehyde with 4 atoms and the largest molecules have 41 atoms. The molecules contain only carbon, oxygen, nitrogen and hydrogen, and frequently have more oxygen than carbon atoms and a maximum of two nitrogen atoms (See Fig. 1d). Finally, *p*_*Sat*_ is approximately normally distributed on a log10-scale ranging from 10^−14^ to 10^6 ^Pa.

**Fig. 2:**
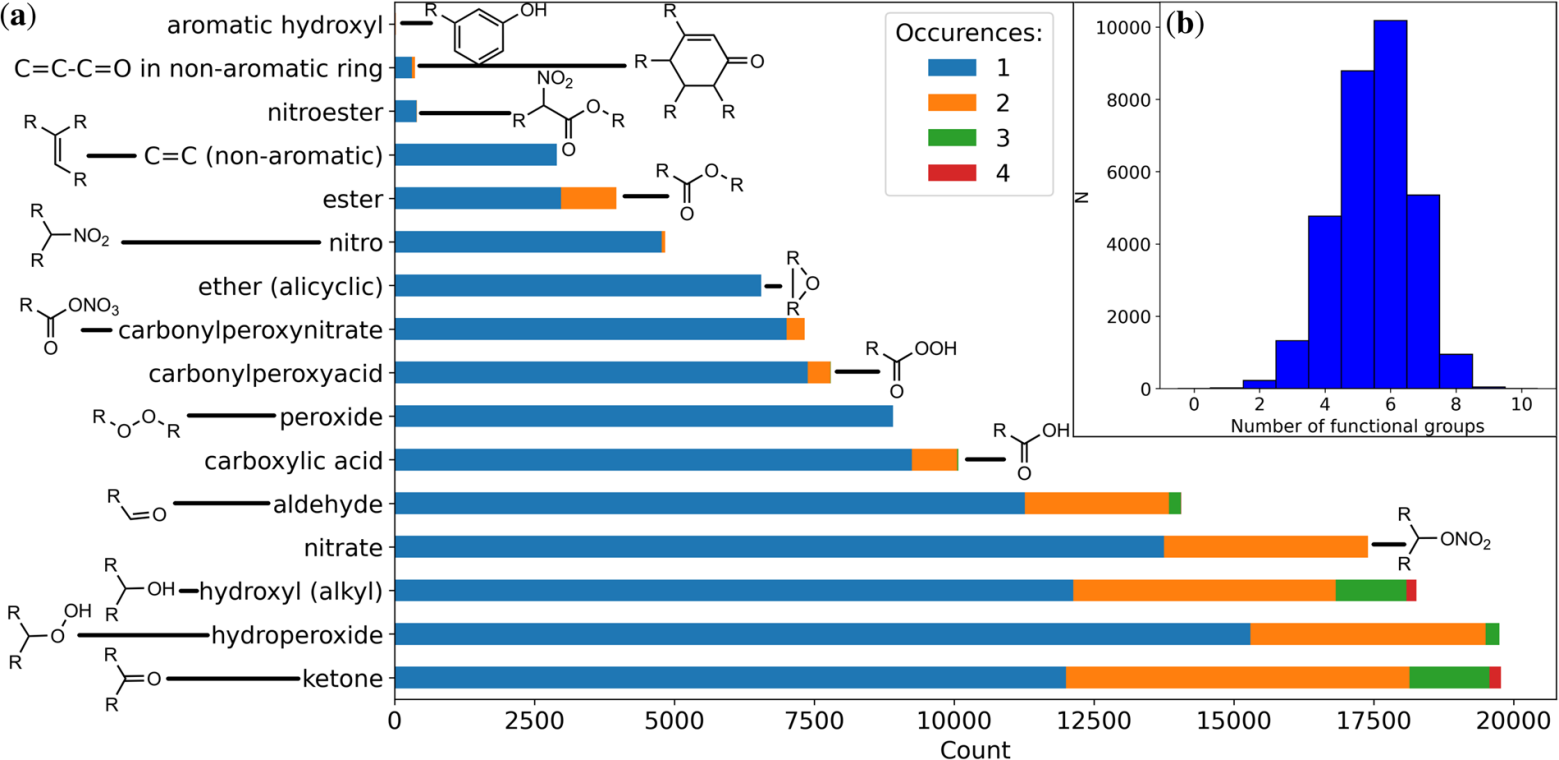
Number and type of functional groups in the data: (**a**) The frequency of occurrence of functional groups per molecule. Four molecules with five ketone groups and six molecules with five hydroxyl (alkyl) groups are not depicted (for clarity). (**b**) A histogram of the number of functional groups per molecule.

Next, we review the frequency and type of functional groups in the dataset. Figure 2 provides an overview of all functional groups in the dataset, as detected by the APRL-substructure finder^32^. The most common groups, hydroperoxides, ketones and hydroxyls, have a large impact on *p*_*Sat*_ as they increase the molecule’s ability to engage in intermolecular interactions in the liquid phase. Generally, a large number of functional groups correlates with a low *p*_*Sat*_. These relationships will be explored in more detail in the *Technical validation* section. The molecules in the GeckoQ dataset have a median of five functional groups, usually more than three and fewer than eight groups.

Our objective was to compile the structural and thermodynamic properties of LVOCs together into a single dataset of high accuracy. Studying the relationship between these properties is necessary to understand the behaviour of molecules in the atmosphere based merely on their structure. The GeckoQ dataset can be used to train machine learning models in atmospheric science, for studying particle formation processes and the role of different conformers in these. Here, we demonstrate such models with Gaussian process regression and the topological fingerprint descriptor. Beyond that, GeckoQ can be used for data-driven studies in (organic) chemistry. We anticipate that GeckoQ will faciliate new atmospheric research like the QM9^33^ or OE62^34^ datasets did in chemistry and materials science.

## Methods

### Dataset curation

The dataset curation involves the selection of relevant molecules and performing quality checks for duplicates, and outliers. We used an initial set of molecules^27^ generated with the GECKO-A^28^ program from the parent species *α*-pinene, decane and toluene. These three were chosen to ensure that GeckoQ covers a diverse range of atmospheric compounds. *α*-pinene is the main monoterpene emitted by vegetation. Monoterpenes are a major source of biogenic emissions and prevalent in large enough concentrations to drive NPF. Conversely, toluene and decane are examples for anthropogenic aromatic and aliphatic emissions.

In our dataset composition, we removed molecules for which the in GECKO-A implemented group contribution method SIMPOL^35^ expected lower *p*_*Sat*_ than 10^−8^ Pa, since they likely react only in the condensed phase, whereas GECKO-A only includes gas phase reactions^27^. We further note that autoxidation reactions, which can form very low-volatility products also in the gas phase, are currently missing from GECKO-A. While the specific structures of autoxidation products are likely to differ somewhat from the molecules included in the GeckoQ dataset, they still contain the same types of functional groups, albeit with a larger percentage of peroxides and hydroperoxides. This limits the possible *p*_*Sat*_ range and the chemical phase space of GeckoQ, but ensures chemical consistency because all data stems from modelling well-known reaction types in the gas phase.

We next refined the list of of 180k molecules and corresponding SMILES strings GECKO-A produced^36^. We removed duplicates that we identified at two stages of data processing. First, we purged 33,827 duplicates based on their SMILES strings in the initial GECKO-A output. Then we removed molecules with identical topological fingerprint (TopFP) descriptors. Of these, 3870 molecules were structural duplicates and redundant, but 223 molecules did exhibit structural differences. We nonetheless chose to remove this small group of molecules to avoid descriptor ambiguities in the future.

We furthermore removed molecules with three or more nitrogen atoms. In GeckoQ nitrogen atoms only occur in the context of nitrate and nitro groups. These groups have only a small effect on *p*_*Sat*_ despite their large mass and are thus less interesting for particle formation. From the remaining 157k molecules, we randomly selected 31,640 for COSMO*conf* and COSMO*therm* calculations.

Further, we inspected all molecules with a *p*_*Sat*_ lower than 10^−13^ and higher than 10^4 ^Pa for outliers. In three cases we found that the molecular structure and calculated *p*_*Sat*_ are inconsistent with each other, because we expected a different *p*_*Sat*_ based on the molecular structure, i.e we found molecules that only differed by a single functional group but had a vastly different *p*_*Sat*_ (the contribution of a single functional group to the *p*_*Sat*_ cannot be arbitrarily large).

We removed the three molecules from the data, resulting in an overall dataset of **31,637** molecules.

### Computation of thermodynamic properties

We focused on atmospherically relevant *thermodynamic properties*. We computed *p*_*Sat*_ [Pa] and the heat of vaporisation [kJ/mol] which are related to the equilibrium between the liquid and the gas phase and therefore describe the likelihood of a molecule to contribute to particle formation and growth. We also calculated the chemical potential [kJ/mol] in the liquid of each molecule and the “free energy of a molecule in mixture” [kJ/mol]. The calculation required a set of conformer structures optimized for the liquid phase and a corresponding set optimized in the gas phase for each molecule. We included the liquid environment implicitly using the Conductor-like Screening Model for Real Solvents^37,38^ (COSMO-RS), which is a continuum solvation model. The final thermodynamic properties were calculated with COSMO*therm*^37,38^ taking all aforementioned molecular conformers into account.

**Fig. 3:**
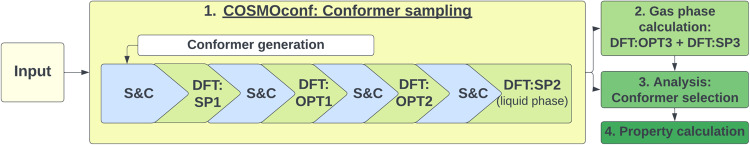
Workflow for the data label calculation. “S&C” stand for a step of clustering and sorting the molecules, “DFT:SP” is a single point DFT calculation, and “DFT:OPT” stands for a DFT structure optimisation.

We managed the calculations for GeckoQ with the workflow manager Merlin (https://merlin.readthedocs.io). For each molecule, we carried out the four steps illustrated in Fig. 3. Each step is described in detail in the following.

#### Conformer sampling and refinement

We used COSMO*conf* (www.3ds.com/products-services/biovia/) to find the low energy conformers for each molecule. The full COSMO*conf* “job template” is provided in the GeckoQ data repository and contains technical details on all steps. “Input” (cf. Fig. 3) to COSMO*conf* is one arbitrary 3-dimensional structure generated from the molecular SMILES string with the BALLOON^39^ conformer generator. Initially, the COSMO*conf* program executed a conformer search: It generated and optimized 10,000 conformers employing the “distance geometry” method^40^ implemented in rdkit^41^ (energy threshold 200 kcal/mol, RMSD threshold 0.0^41^) and it generated an additional 600 conformers with the genetic algorithm implemented in BALLOON^39^ (with default parameter found in the COSMO*conf* user guide 2021 p. 39) optimizing them with the MMFF94^42^ force field.

Most of the generated conformers were structurally similar and were removed with COSMO*conf*’s “CLUSTER_ GEOCHECK” and “CLUSTER_MU” routines, clustering them according to their geometry and energy, respectively, and removing non-unique structures (“S&C” in Fig. 3). The “CLUSTER_GEOCHECK” routine maps conformer structures onto each other. If mapped atom types are different, or if the weighted local similarity measure between all atoms exceeds a threshold of 0.5 Å or 20°, then the conformers are different. Further details can be found in the COSMO*conf* manual. Secondly, “CLUSTER_MU” clusters conformers with respect to their chemical potential in mixture, where conformers with a potential difference larger than 0.2 kcal/mol are considered different.

All DFT calculations were carried out with Turbomole^43^ using the multipole accelerated RI-approximation^44^ and employed the Becke-Perdew (BP86)^45,46^ exchange-correlation functional. To save computational time, the conformer search was hierarchically structured as displayed in Fig. 3. With a cheap SV(P) basis set the DFT energy of all conformers generated with COSMO*conf* was calculated (DFT:SP1). This set was reduced in a clustering and sorting step (“S&C” in Fig. 3). The geometry of the remaining conformers was optimized with the same basis set (DFT:OPT1). A subsequent S&C step reduced the conformer set further. To increase the accuracy, we repeated the geometry optimization with a tighter def-TZVP basis set (DFT:OPT2). The final energy was calculated with the def2-TZVPD basis set (DFT:SP2). All of these calculations involve the COSMO-RS^37,38^ model, providing a discrete charged surface surrounding the molecule for each conformer, and we will refer to them as “liquid phase conformer”. This surface is utilized later by COSMO*therm*. The gas phase conformers were obtained from the liquid phase conformers by repeating the geometry optimization of each liquid phase conformer, but without the implicit solvation model. We again performed the geometry optimization with a slightly cheaper basis set (TZVP; DFT:OPT3) than the final energy calculation (TZVPD; DFT:SP3).

#### Conformer selection and property calculation

Because COSMO*therm* overestimates the impact of intramolecular H-bonds^29^, we selected only the conformers with a minimal number of these H-bonds for calculating the thermodynamic properties. First, we performed an initial COSMO-RS calculation with the *pr_steric* keyword, which identifies the number of intramolecular H-bonds for each conformer. We proceeded to choose the energetically lowest conformers with zero intramolecular H-bonds up to a maximum of 40 conformers, following the example of previous work^30^. If there were no conformers with zero intramolecular H-bonds, we chose conformers with one intramolecular H-bond, and if there were none of those, two, or three.

We utilized the selected conformers to compute thermodynamic properties with COSMO*therm*^37,38^. In COSMO*therm* the *p*_*Sat*_ is calculated for each single conformer of a molecule with the assumption of a pure “solvent” consisting of that same conformer. The solvent is constructed with the discrete charged surfaces provided by the COSMO-RS DFT calculations. All the conformer *p*_*Sat*_ are then weighted according to their overall population, which is determined by the Boltzmann distribution of states with different free energies, resulting in a single *p*_*Sat*_. The calculations were conducted at a standard temperature of 298.15 K. The files we provide allow for a re-calculation of the properties at a different temperature (See section *Usage notes*).

### SIMPOL

The *p*_*Sat*_ of a molecule can also be computed, e.g. with the group contribution method SIMPOL^35^, which is frequently employed by the atmospheric research community^21,27,30^. As a form of validation, we compare the DFT *p*_*Sat*_ to those of SIMPOL *p*_*Sat*_ (cf. *Technical Validation* section). SIMPOL is based on

1 $${{\rm{\log }}}_{10}{p}_{Sat}=\sum _{k}{\nu }_{k}{b}_{k},$$

where *v*_*k*_ is the number of functional groups of type *k* found in a molecule and *b*_*k*_ is a group-specific parameter that has been fitted to reference data. The APRL Substructure Search Program (APRL-SSP)^32^ is the only publicly available program that can extract SIMPOL *v*_*k*_’s from SMILES strings (cf. Fig. 2). We found that APRL-SSP does not count carbonyl groups attached to a carbon that is also attached to a peroxy group, and corrected the number of ketones and aldehydes accordingly. After correction, we calculated *p*_*Sat*_ with our own Matlab SIMPOL implementation.

### Structural descriptor: topological fingerprint

For machine learning, molecules need to be represented in a machine readable format, a so-called *descriptor*^47^. In previous work^25^, some of us had investigated a variety of molecular descriptors for learning atmospherically relevant thermodynamic properties: the Coulomb Matrix^48^, the Many-Body-Tensor-Representation (MBTR)^49^, the MACCS structural key^50^, the Topological Fingerprint (TopFP)^41,51^, and the Morgan Fingerprint^41,52^. MBTR and TopFP provided the highest accuracy for a kernel-ridge regression based model^25^. For the machine-learning model of our GeckoQ data, we therefore chose the TopFP, because it produces the same descriptor for all conformers of a molecule and is thus not sensitive to the precise atomic structure of the GeckoQ molecules. Additionally, it is computationally inexpensive compared to MBTR. The TopFP hyperparameters had to be optimised and adjusted to the current dataset. The optimized hyperparameters we found were a size of 8192 for the descriptor array, a minimum path of 1, a maximum path of 9, and 6 bits per hash.

### Gaussian process regression

The GeckoQ dataset is intended to facilitate the application of machine learning methods in the field of atmospheric research. To demonstrate a first use case, we employ Gaussian process regression (GPR), a kernel-based probabilistic tool for supervised machine learning^53^, to predict the *p*_*Sat*_ of molecules from their geometry.

In GPR, a prior belief of the outcome is combined with the data in Bayes’ rule to perform the regression and compute the GP posterior. The mean of the GP posterior constitutes the prediction and its variance is a measure for the reliability of the result. The model covariance is encoded into a kernel function. We deployed an uninformative GP prior and a product kernel, where a constant *θ*_*s*_ multiplies the Radial Basis Function (RBF) kernel:

2 $${K}_{{\rm{RBF,s}}}\left({x}_{1},{x}_{2}\right)={\theta }_{s}\ast \exp \left(-\frac{1}{2}{\left({x}_{1}-{x}_{2}\right)}^{{\rm{T}}}{\theta }_{l}^{-2}{\left({x}_{1}-{x}_{2}\right)}^{{\rm{T}}}\right).$$

The kernel function contains the signal lengthscale *θ*_*l*_ and the function amplitude *θ*_*s*_ as hyperparameters. We optimized *θ*_*l*_ and *θ*_*s*_ by maximizing the negative log marginal likelihood during data fitting, which is equivalent to conducting a global search in the hyperparameter space. To avoid local minima, we restarted each log marginal likelihood maximization six times. The resulting *θ*_*l*_ and *θ*_*s*_ values lie both in the range of 2000 to 6000, depending on the training data size. The data noise was also treated as a hyperparameter and optimized consistently to a value of 0.23. We used the Pytorch python package^54^ for all GPR calculations. Since *p*_*Sat*_ varies across many orders of magnitude, we learned the log10 *p*_*Sat*_. All data were normalized prior to machine learning.

To assess GPR performance, we divided the data into two subsets, a *test set* and a *training set*. We calculated the mean average error (MAE) between the predictions and the actual *p*_*Sat*_ as a measure of accuracy. The MAE was chosen in continuity with previous work^25^. To assess learning success, we computed a learning curve by training a series of GP models for training set sizes from 2000–28,000 molecules in steps of 2000 and evaluated the models with a testset of 2000 molecules. We applied 5-fold cross validation, to obtain five separate models, and averaged the resulting MAEs, to account for statistical fluctuations.

## Data Records

The main GeckoQ dataframe (Dataframe.csv) consists of 31,637 rows with entries for the identifiers, attributes, labels and functional groups for each molecule. Table 1 provides a detailed break-down of the Dataframe.csv. For completeness, we also included the topological fingerprints and the RDkit objects for each molecule in GeckoQ. The corresponding TopFP and RDkit objects are stored in separate files, TopFP.jl and RDkitObjects.jl, respectively, and are labelled according to the index of the molecules.

**Table 1:** Detailed description of all the columns in the *Dataframe.csv* file.

No.	Column name	Unit	Description
1	index	—	A unique molecule index used in naming files, see in Table 2.
2	SMILES	—	The canonical SMILES string as provided by GECKO-A.
3	InChIKey	—	The standard InChIKey of the molecule.
4	pSat_Pa	Pa	The *p*_*Sat*_ of the molecule calculated by COSMO*therm*.
5	ChemPot_kJmol	kJ/mol	The chemical potential of the molecule calculated by COSMO*therm*.
6	FreeEnergy_kJmol	kJ/mol	The free energy of the molecule calculated by COSMO*therm*.
7	HeatOfVap_kJmol	kJ/mol	The heat of vaporisation of the molecule calculated by COSMO*therm*.
8	MW	g/mol	The molecular weight of the molecule.
9	NumOfAtoms	—	The number of atoms the molecule.
10	NumOfC	—	The number of carbon atoms the molecule.
11	NumOfO	—	The number of oxygen atoms the molecule.
12	NumOfN	—	The number of nitrogen atoms the molecule.
13	NumHBondDonors	—	The number of hydrogen bond donors in the molecule i.e. hydrogens bound to a oxygen.
14	NumOfConf	—	The number of stable conformers found and successfully calculated by COSMO*conf*.
15	NumOfConfUsed	—	The number of conformers that has been used to calculate the thermodynamic properties. The selection of these conformers is discussed more detailed in Sec. *Conformer selection and property calculation*.
16	parentspecies	—	Either “decane”, “toluene”,“apin” for *α*-pinene, or a combination of these connected by an underscore to indicate ambiguous descent. In 243 cases the parent species is “None”, because it was not possible to retrieve it.
17	C = C (non-aromatic)	—	The number of non-aromatic C = C bounds found in the molecule.
18	C = C-C = O in non-aromatic ring	—	The number of C = C-C = O structures found in non-aromatic rings in the molecule.
19	hydroxyl (alkyl)	—	The number of the alkylic hydroxyl groups found in the molecule.
20	aldehyde	—	The number of aldehyde groups found in the molecule.
21	ketone	—	The number of ketone groups found in the molecule.
22	carboxylic acid	—	The number of carboxylic acid groups found in the molecule.
23	ester	—	The number of ester groups found in the molecule.
24	ether (alicyclic)	—	The number of alicyclic ester groups found in the molecule.
25	nitrate	—	The number of nitrate groups found in the molecule.
26	nitro	—	The number of nitro groups found in the molecule.
27	aromatic hydroxyl	—	The number of aromatic hydroxyl groups found in the molecule.
28	carbonylperoxynitrate	—	The number of carbonylperoxynitrate groups found in the molecule.
29	peroxide	—	The number of peroxide groups found in the molecule.
30	hydroperoxide	—	The number of hydroperoxide groups found in the molecule.
31	carbonylperoxyacid	—	The number of carbonylperoxyacid groups found in the molecule.
32	nitroester	—	The number of nitroester groups found in the molecule.

GeckoQ includes 7,259,598 conformer structures. Dataframe_conformerE.csv contains liquid phase and gas phase energies for all conformers. These energies were extracted from each conformer’s “.cosmo” file (liquid phase conformer) and the “.energy” file (gas phase conformer), that are also available. In addition, we collected various input, output and intermediate files generated in the label calculation process and compressed them in a separate zip archive for each molecule. The different file types are explained in Table 2. We have further grouped every 800 hundred molecules in a tar archive. A list of the resulting 40 tar archives and their molecular indices is provided in the README.md of the data repository.

**Table 2:** Details to all the files that can be found in the data repository for each molecule.

file name	type	description
*$id*.sdf	structure	A structure created from SMILES strings and a input file of COSMO*conf* for molecule *$id*.
*$id*_c*$i*.cosmo	structures	The COSMO*conf* output file. Contains the structure and energy for the liquid phase of conformer *$i* of molecule *$id*. Conformers are ranked according to rising energy (“Total energy [a.u.]”) and *$id*_c0.cosmo is the most stable conformer. In some cases, some conformers were removed due to computational errors or non-convergence.
*$id*_c*$i*.energy	structures	The structure and energy file for the gas phase conformers of molecule *$id*.
*$id*-h-bonds.inp	input	The input file for the *pr_steric* calculation to determine the number of intramolecular hydrogenbonds for each conformers. It accepts an input file with the list of all conformers, which can be reconstructed from the entry “*$id*-h-bonds-confs.txt”.
*$id*-h-bonds.out	output	The output file of the *pr_steric* calculation. It contains electrostatic and steric information for each conformer. It is possible to retrieve the number of intramolecular H-bonds by checking the overlap of donor groups with neighbouring acceptor groups.
*$id*-h-bonds-confs.txt	list	A list of all conformers and corresponding numbers of “partial” H-bonds and “full” H-bonds.
COSMOFILES-lt*$MinHBonds*bonds.txt	list	A list of all conformers with a minimum number of H-bonds, *$MinHBonds*. Details in Section *Property calculation*. This file is required by the COSMO*therm* calculation input.
lt*$MinHBonds*bonds.inp	input	The input file for the COSMO*therm* calculation using only conformers with *$MinHBonds* H-bonds.
lt*$MinHBonds*bonds.out	output	The output file for the COSMO*therm* calculation using only conformers with *$MinHBonds* H-bonds. It contains all the thermodynamic labels we calculate.

The file names contain variables where *$id* refers to the entry of the molecule in the “index” column, *$i* is the number of a conformer and *$MinHBonds* is the minimum number of H-bonds found for any conformers of a molecule. The “structure” type are files that contain a molecular 3d xyz structure.

GeckoQ is freely available for download from its Fairdata.fi Etsin repository: 10.23729/022475cc-e527-41a9-bbc0-0113923cf04c^55^. The data is organised as follows:

- README.md: General information regarding the data, also provided in this section, “Usage Notes”, and “Figures and Tables”.
- Dataframe.csv: Properties and attributes of the GeckoQ molecules (see Table 1).
- Dataframe_conformerE.csv: Liquid phase (“totE_liq” column) and gas phase energies (“totE_gas” column) of the GeckoQ conformers.
- TopFP.jl: The index and the topological fingerprint for each molecule in GeckoQ for machine learning.
- RDkitObjects.jl: A data frame with the indices and an rdkit object for each molecule to facilitate quick and simple visualization, or calculation of different attributes and descriptors.
- Data entries/: 40 tar-archives each with 800 zip files, one for each molecule, containing the files specified in Table 2.
- Code/: A directory with a jupyter notebook containing instructions on how to load, transform and visualize the data, and with a bash script containing instructions on handling the data files (see Sec. “Usage Notes”). The directory also contains the applied COSMO*conf* job template.

## Technical Validation

To validate GeckoQ, we review the applied *p*_*Sat*_ calculation procedure to check for convergence and for physical and chemical consistency. In addition, we show that the computed *p*_*Sat*_ are consistent with simpler models, and demonstrate a first machine learning application.

First we survey the computational uncertainty of the COSMO-RS model applied in the combined COSMO*conf* and COSMO*therm* approach. The COSMO-RS model was originally parametrized with experimental values of 217 molecules^38^ and later refined with another 310 molecules^56^. These reference molecules include a diverse range of organic molecular classes and contain the elements H, C, N, O, and Cl with F, S, Br and I added in the refinement. The following accuracies were reported: maximum of 0.566 log units (vapor pressures), 0.451 log(max/*γ*^∞^) (activity coefficients), and 1.2 kJ/mol (Gibbs free-energies). Relative to the *p*_*Sat*_ range of twenty orders of magnitude in GeckoQ, the expected COSMO vapor pressure error and the inherent data noise *σ* of 0.23 log(*σ*/Pa) estimated by GPR are in good agreement. Further, we review the quality of our COSMO*conf* settings. In the initial conformer search for the generation of GeckoQ data we applied the most accurate settings that COSMO*conf* offers. We utilized the BALLOON program as well as rdkit for conformer generation to ensure that we captured all relevant structures. We furthermore conducted the final DFT calculations at the highest fidelity level included in COSMO*conf* and COSMO*therm* (BP86/def2-TZVPD). We also monitored resulting conformer structures. Failure of conformer calculations to complete correctly (resulting in unphysical energies), or dissociation of a conformer during the structure optimization was indicated by warnings in the COSMO*therm* output. In such cases, we removed the few conformers in question and repeated the thermodynamics calculation with all the remaining conformers.

**Fig. 4:**
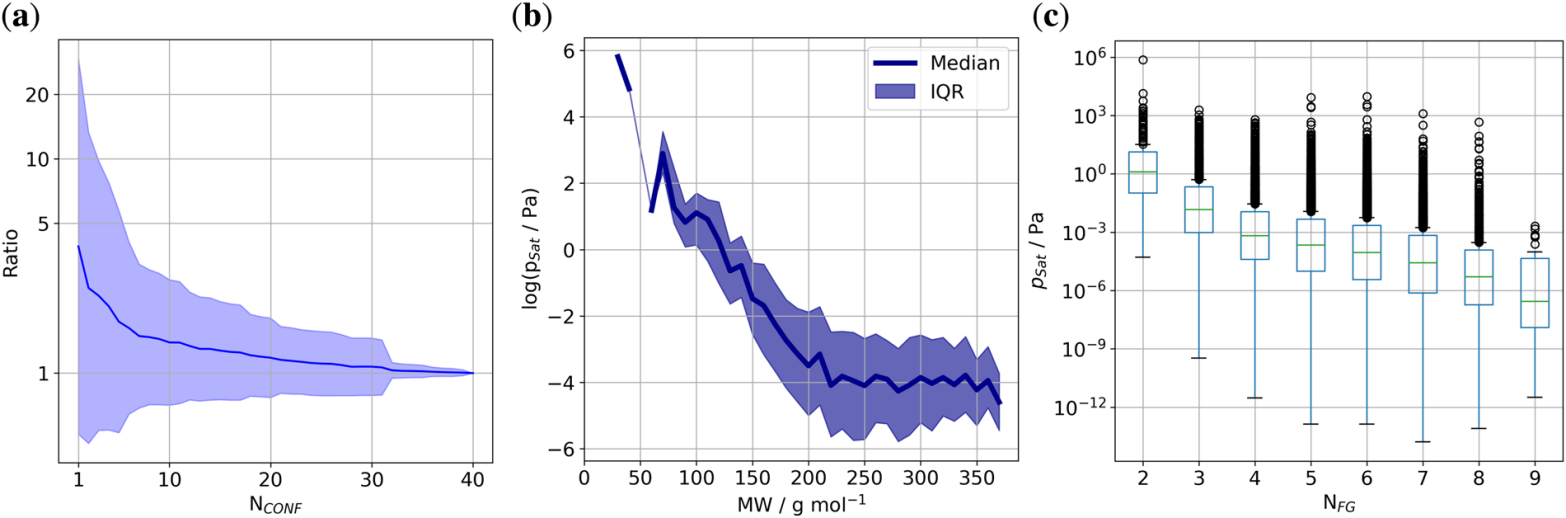
The relationship of *p*_*Sat*_ with number of conformers N_*CONF*_, molecular weight MW, and number of functional groups N_*FG*_: (**a**) The change of *p*_*Sat*_ with the number of conformers. *Ratio* is the calculated *p*_*Sat*_ at N_*CONF*_ = 40 divided by *p*_*Sat*_ at N_*CONF*_ in log10 scale. (**b**) Log10 of *p*_*Sat*_ median and interquartile range (IQR) plotted against the molecular weight MW binned into bins of 10 g/mol. (**c**) Boxplots of *p*_*Sat*_ by the number of all found functional groups in one molecule.

As noted in Section *Conformer selection and property calculation*, *p*_*Sat*_ is obtained from a weighted average over multiple conformers. For this reason, we checked how sensitive the value of *p*_*Sat*_ is to changes in the conformer selection. We considered all molecules with at least 40 conformers, then randomly chose a subset of 110 for this test. First, the *p*_*Sat*_ was calculated using only the most stable conformer, then we added the next most stable conformer to the selection and computed the result again. This was repeated until we reached 40 conformers. For each of the interim *p*_*Sat*_ values, we computed the ratio to the *p*_*Sat*_ obtained with the maximum number of 40 conformers. Figure 4a displays the mean and standard deviation for this ratio averaged over all 110 molecules. The figure illustrates that the *p*_*Sat*_ for a single conformer deviates by a factor of 3.9 from the converged result. As more conformers are added, this discrepancy decreases and converges to a 1:1 ratio at 32 conformers. The drop of the standard deviation at 32 conformers is caused by a single outlier, where the addition of the 32nd conformer changed the vapor pressure ratio by 1.3 order of magnitude. Based on the average of these 110 molecules, we conclude that the *p*_*Sat*_ values in the GeckoQ data are not sensitive to conformer numbers higher than 32. Thus we can confirm that our choice of a maximum number of 40 conformers was adequate for good precision of *p*_*Sat*_. For molecules with fewer conformers than 32, all conformers were included.

Next, we examine the relationship between the COSMO*therm p*_*Sat*_ and molecular structural properties. We focus on the molecular weight (MW) as a universal measure for molecule size and functional groups, since functional groups have the largest influence on *p*_*Sat*_. For example, functional groups can establish **inter**molecular as well as **intra**molecular interactions. Intermolecular interactions lead to a stabilization of the molecule in the liquid phase, i.e a low *p*_*Sat*_, whereas intramolecular interactions stabilize the molecule in the gas phase and lead to a high *p*_*Sat*_.

In Fig. 4b we plotted *p*_*Sat*_ as function of the MW. For small molecules *p*_*Sat*_ is high. It decreases with increasing MW before leveling out at approximately 220 g/mol and a *p*_*Sat*_ of roughly 10^−4^ Pa. The decrease of *p*_*Sat*_ is consistent with Fig. 4c that shows that a higher number of functional groups decreases *p*_*Sat*_. Beyond 220 g/mol the abundance of nitrate (62 g/mol) and nitro (46 g/mol) groups (see Fig. 2) dominates (The largest molecules without any nitrate- or nitro- groups have a MW of 282 g/mol). These groups have a comparatively large mass, but their contribution to a lower *p*_*Sat*_ is small, which explains the saturation of *p*_*Sat*_ to a low value. Note also, that during dataset curation, molecules with very low saturation vapor pressure were removed, which provides another reason for *p*_*Sat*_ leveling out with increasing MW.

Next we present the SIMPOL consistency check. Extraction of the functional groups for SIMPOL is not a trivial problem. The molecules in GeckoQ contain a large number and high density of functional groups. To verify APRL-SSP’s extraction accuracy including our correction for “carbonyl-peroxides”, we visually inspected 100 randomly chosen molecules, ensuring that all possible functional groups were present. All functional groups that we found were also found by APRL-SSP. However, we cannot exclude the possibility that combinations of functional groups (such as the “carbonyl-peroxide” group) exist that make the results unreliable, albeit very infrequently. Overall, we report an accuracy of more than 99% for the GeckoQ functional groups.

**Fig. 5:**
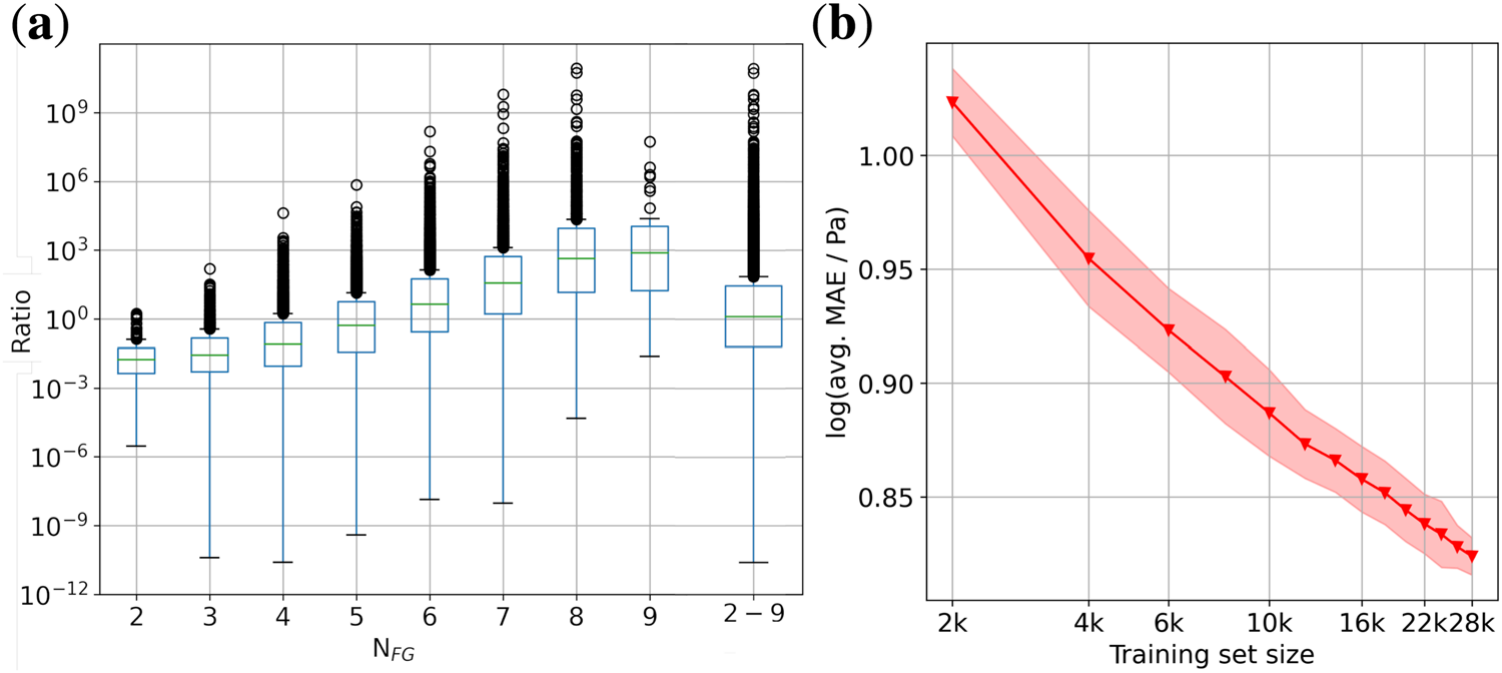
Comparison of *p*_*Sat*_ obtained by SIMPOL and predicted with GPR: (**a**) The ratio of the COSMO*therm p*_*Sat*_/SIMPOL *p*_*Sat*_ vs. the number of functional groups. Additionally, the ratio for the sum of 2 to 9 functional groups. (**b**) Learning curve produced with Gaussian Process Regression. The data points are the mean MAEs of 5 training runs where the coloured area is the mean ± standard deviation.

Figure 5a shows the ratio of the COSMO*therm p*_*Sat*_ to SIMPOL’s as a function of the number of functional groups *N*_*FG*_ in the corresponding molecules. Ideally, the median would lie around one, but we find it to increase with *N*_*FG*_. For molecules with 2–5 functional groups, the SIMPOL *p*_*Sat*_ is higher than COSMO*therm*’s. The behaviour reverses for 6–9 functional groups. This trend is consistent with the difference between SIMPOL and COSMO*therm*. Both methods account for **inter**molecular interactions, but only COSMO*therm* accounts also for **intra**molecular interactions, which lead to a higher *p*_*Sat*_. **Intra**molecular interactions become more important for large *N*_*FG*_, for which we observed the ratio reversal and larger deviation between SIMPOL and COSMO*therm* in line with previous such comparisons^30,57^.

Finally, we applied a GPR to the GeckoQ data to map the molecular structures to *p*_*Sat*_. The resulting learning curve is depicted in Fig. 5b. The MAE for the minimal training set size of 2k is 1.02 log(MAE/Pa). With more training data, the error reduces to 0.82 log(MAE/Pa) at a training set size of 28k. The learning rate is not constant. For small training sets, the GPR learns slightly faster than for larger, where we suspect that our machine-learning model finds less diversity and more redundancy in GeckoQ.

The final MAE 0.82 log(MAE/Pa) at 28k is similar to the data uncertainties reported for COSMO*therm* of 0.5 log units^56^, and the inferred data noise of 0.23 log(*σ*/Pa). This error is smaller than the average deviation between SIMPOL and COSMO*therm*. The MAE of the GPR could be further reduced with more data. By extrapolating the learning curve, we estimate that a training set with 2 mio. molecules would be needed to bring the MAE down to 0.5 log units.

Previous work^25^ also employed the TopFP descriptor in a kernel ridge regression machine learning model to learn *p*_*Sat*_ as a function of atomic structure for a different atmospheric dataset. They obtained a MAE of 0.31 log(MAE/Pa). Their dataset contained a comparatively narrow range of molecules, with a median of *N*_*FG*_ = 3 and a range of 1–6 functional groups. The GeckoQ molecules are larger, more complex and more diverse and thus harder to learn, which manifests in a higher MAE^26^.

## Usage Notes

The Etsin repository with the data contains the directory Code/ with a jupyter notebook BasicDataprocessing.ipynb. The notebook includes basic instructions on how to load, analyze, and search the data, and how to transform the data to a descriptor and target array for machine learning. Further, it also can be a guide for using the rdkit toolbox to calculate molecule properties or to create new descriptors, and additional contains our correction for the counts of ketone and aldehyde groups. The jupyter notebook is also provided as html file. The Dataframe.csv and Dataframe_conformerE.csv can be loaded by any programming language for statistical computing, whereas TopFP.jl and RDkitObjects.jl need to be loaded with the joblib python package.

When machine learning methods are applied to labels such as the *p*_*Sat*_, we recommend transforming the values to their log10, because machine learning algorithms are usually sensitive to the scale of the data. Code/ also contains the bash script FileProcessing.sh with code for processing the conformer files or COSMO*therm* output. It also demonstrates how to unzip only the single energetically most stable conformers or the fast extraction of values from the COSMO*therm* output. Moreover, FileProcessing.sh contains all steps for recalculating a molecules properties for a different temperature.

## Data Availability

Custom code written for data generation mainly consists of scripts for pre- and postprocessing steps linking together the software mentioned below. These scripts are executed through a Merlin workflow. All these scripts are publicly available in a GitHub repository: https://github.com/Supervitux/COSMO_on_Merlin^58^. GECKO-A is available at their website http://geckoa.lisa.u-pec.fr/. COSMO*conf* 4.3 and COSMO*therm* 2021 and their licenses were purchased from Dassault Systemes (https://www.3ds.com/). We provide our custom COSMO*conf* jobtemplate ((COSMOConfProtocol.xml in the repository. Merlin version 1.7.5 is freely available from https://merlin.readthedocs.io/en/latest/index.html#.
